# The Intestinal Fate of Phenolic Substances in Honey: Metabolism by Gut Microbiota, Regulation of Bioavailability, and Its Prebiotic Effects on Host Health

**DOI:** 10.3390/foods15152720

**Published:** 2026-08-02

**Authors:** Qiao Yang, Wenna Yu, Tongyi Wang, Xiangxin Li, Renpeng Du

**Affiliations:** 1 Engineering Research Center of Agricultural Microbiology Technology, Ministry of Education & Heilongjiang Provincial Key Laboratory of Plant Genetic Engineering and Biological Fermentation Engineering for Cold Region & Key Laboratory of Microbiology, College of Heilongjiang Province & School of Life Sciences, Heilongjiang University, Harbin 150080, China2231660@s.hlju.edu.cn (W.Y.); 2251662@s.hlju.edu.cn (T.W.); 2 State Key Laboratory of Resource Insects, Institute of Apiculture Research, Chinese Academy of Agricultural Sciences, Beijing 100093, China

**Keywords:** honey, phenolic substances, gut microbiota, prebiotic, gut–target axis, precision nutrition

## Abstract

This review systematically summarizes the metabolic transformation patterns, bioavailability regulation mechanisms, and multi-target host health effects of honey phenolic compounds under the action of gut microbiota, based on literature from 1996 to 2026. It focuses on elucidating the “high efficacy in vitro, low exposure in vivo” paradox, the three-step cascade metabolic pathway (deglycosylation, C-ring cleavage, and reduction with secondary transformation), and the synergistic prebiotic effects of “oligosaccharides plus phenolics.” Current evidence indicates that the health benefits of honey are largely derived from microbiota-derived small-molecule metabolites rather than from the parent compounds, and these metabolites exert regulatory effects via the gut–liver, gut–brain, gut–immune, and gut–fat axes. However, the vast majority of evidence comes from in vitro fermentation, animal models, or ex vivo fecal cultures, with a severe shortage of high-quality human intervention studies, thereby limiting causal inference. Therefore, this review proposes a shift in honey quality evaluation from traditional qualitative chemical composition-based assessment toward precision functional evaluation based on metabolite profiles, and emphasizes the urgent need to establish standardized metabolite-based quality markers, develop enterotype-based stratified nutrition strategies, and conduct rigorous randomized controlled trials, so as to promote the transformation of honey from a traditional food into a targeted dietary intervention agent.

## 1. Introduction

Honey is a natural food and medicinal product with a long history of use. Beyond providing basic dietary energy, its diverse physiological health benefits have been widely recognized, and the core bioactive components are largely attributed to its endogenous phenolic constituents, including phenolic acids and flavonoids [1]. These phenolic compounds have demonstrated notable antioxidant and anti-inflammatory activities in vitro, which have long been considered the primary basis for the health-promoting properties of honey [2]. However, a notable discrepancy has emerged from in vivo pharmacokinetic studies: after oral consumption, the plasma steady-state concentrations of intact phenolic precursors remain at the nanomolar level, substantially lower than the micromolar concentrations required for in vitro efficacy [3]. This observation raises the possibility that the health benefits of honey phenolics may not derive primarily from the direct actions of their parent molecules, but rather rely on indirect pathways involving the gut microbiota [4,5]. The intestinal microbiota, through its extensive enzymatic capacity, can convert dietary phenolic glycosides into small-molecule phenolic acids and short-chain fatty acids, which exhibit altered bioavailability and distinct bioactivity profiles compared to their precursors [6]. These microbial metabolites have been shown to exert hepatoprotective and neuroprotective effects via systemic circulation [7,8]. Thus, understanding the gut microbiota-dependent metabolic fate of honey phenolics is relevant for interpreting their in vivo biological functions.

The gut microbiota is recognized as a key participant in host metabolism, immune modulation, intestinal barrier maintenance, and the biotransformation of dietary xenobiotics [9]. For terminological clarity, we adopt the consensus definitions established by the International Scientific Association for Probiotics and Prebiotics (ISAPP): probiotics are defined as “live microorganisms that, when administered in adequate amounts, confer a health benefit on the host,” while prebiotics are defined as “substrates that are selectively utilized by host microorganisms conferring a health benefit.” Honey contains a range of oligosaccharides and phenolic glycosides that can reach the colon intact and serve as substrates for selective microbial utilization [10,11]. Thus, honey may be considered a natural complex matrix containing both prebiotic substrates and phenolic precursors, rather than a purified prebiotic preparation [5,12]. Despite progress in this field, several aspects remain unclear. The range of microbial metabolites produced from honey phenolics has not been fully characterized. The reasons for inter-individual differences in metabolic outcomes, which are linked to gut microbiota composition, are not well understood. Standardized methods for evaluating honey quality based on metabolite profiles are not yet available. In addition, most current evidence comes from in vitro or animal studies, and human data are limited.

This review aims to synthesize the available evidence on these aspects and to outline key unresolved questions for future research. Specifically, we consolidate current knowledge concerning the intestinal metabolism of honey phenolic compounds, the modulation of their bioavailability by gut microbial activity, and the pleiotropic effects of microbial metabolites on host health, with a particular focus on the gut–liver, gut–brain, gut–immune, and gut–fat axes. Through this integrated summary, we seek to facilitate a paradigm shift in the functional evaluation of honey-from a predominantly chemical composition-based assessment toward a more metabolism-oriented framework.

## 2. Biochemical Composition of Honey

Honey is a complex biological matrix containing over 300 to 600 identified chemical constituents. The major components are carbohydrates, accounting for approximately 79–80% of honey’s dry weight, primarily the monosaccharides fructose (30–45%) and glucose (28–40%), along with numerous di- and oligosaccharides such as maltose, turanose, isomaltose, kojibiose, and erlose [13]. Beyond sugars, honey contains a diverse array of minor but biologically active constituents. Proteins and free amino acids (predominantly proline, which accounts for 50–85% of the total amino acid content) are present at approximately 0.5%. Enzymes such as diastase (amylase), invertase (sucrase), glucose oxidase, catalase, and acid phosphatase are also present [14]. Organic acids (including gluconic, citric, malic, and lactic acids), vitamins (particularly B-complex vitamins and vitamin C), and approximately 54 minerals (with potassium, sodium, calcium, and magnesium as the most abundant) further contribute to honey’s compositional complexity [15]. In addition, over 400 volatile compounds have been identified across different honey varieties [16]. Among all these constituents, the phenolic compounds-comprising phenolic acids (such as caffeic acid, p-coumaric acid, and gallic acid) and flavonoids (including quercetin, kaempferol, luteolin, and apigenin)-are of particular interest due to their well-documented antioxidant and anti-inflammatory activities [1]. This rich and varied biochemical composition, which varies significantly with botanical and geographical origin, provides the chemical foundation for the subsequent discussion of honey phenolic metabolism by the gut microbiota.

## 3. Metabolism of Honey Phenolic Compounds in the Intestine and Prebiotic Effects

### 3.1. Core Paradox of In Vitro Activity and Low In Vivo Exposure

Since the 1990s, in vitro antioxidant studies of honey phenolic compounds have formed a system. A large amount of evidence has confirmed that the components of honey phenolic compounds have strong free radical scavenging activity and show significant concentration dependence [17]. The total phenol content of dark honey is generally about 1.5 times or higher than that of light honey, and the corresponding gradient increase in antioxidant capacity is observed [18]. At the anti-inflammatory mechanism level, quercetin can efficiently block the LPS-induced inflammatory cascade reaction of macrophages, target-downregulate the expression of key inflammatory proteins such as iNOS and COX-2, and inhibit the excessive release of pro-inflammatory factors such as TNF-α and IL-6. The molecular mechanism mainly relies on the negative regulation of the NF-κB and MAPK signaling axes [19]. Based on sufficient in vitro activity data, the components of honey phenolic compounds have long been defined as natural, safe, and highly effective antioxidant and anti-inflammatory functional substances.

However, with the deepening of in vivo metabolic research, a core contradiction gradually emerged. After regular dietary intake of honey, the steady-state exposure concentration of phenolic precursors in the plasma of the subjects only remains at the nmol/L level, which is 2 to 3 orders of magnitude lower than the μmol/L concentration required for in vitro efficacy [3]. Taking the typical flavonoid monomer quercetin as an example, its peak plasma concentration after oral administration is lower than 0.5 μmol/L, and the majority of the components in the circulation exist in forms of non-directly physiologically active glucuronide- or sulfate-conjugated metabolites [20,21]. This apparent paradox suggests that the direct systemic actions of intact phenolic precursors are likely limited, and that a substantial portion of their in vivo effects may be mediated indirectly through gut microbiota-dependent pathways [5].

### 3.2. Gut Microbiota: The “Metabolic Engine” of Honey Phenolic Compounds

Traditional food nutrition and pharmacology research mainly focuses on the liver-mediated combined metabolism (glucuronidation, sulfation, methylation), whose products significantly increase polarity and can be rapidly excreted through the liver and kidneys [22]. However, only a small portion of dietary phenolic compounds can be absorbed by the small intestine and enter the liver for metabolism, while the vast majority of unabsorbed phenolic compounds directly reach the colon. During this process, the gut symbiotic microbiota initiates a set of polyphenol skeleton degradation and transformation programs that the host does not possess. Through a series of enzymatic reactions such as glycoside hydrolysis, reduction, ring-opening, decarboxylation, and dehydroxylation, complex polyphenols with low bioavailability are converted into highly active and highly absorbable small molecule metabolites. This gut microbiota-dominated polyphenol transformation process is considered to be a key step in mediating the in vivo physiological functions of dietary phenolic compounds [6].

Most natural honey phenolic compounds exist in the form of glycosides. The dominant gut microbiota highly expresses β-glucosidase and rhamnosidase, which preferentially remove the sugar groups at the terminal of phenolic molecules and release active aglycones. This hydrolysis step is the core limiting step of the deep metabolism of the microbiota [23]. After the aglycones are released, the gut microbiota can specifically cleave the C ring of the flavonoid core and complete directional structural remodeling: quercetin is converted to 3,4-dihydroxyphenylacetic acid (3,4-DHPAA), luteolin is degraded to 3,4-dihydroxyphenylpropionic acid (3,4-DHPPA); the process is accompanied by double bond reduction, aromatic ring opening and hydrolysis reactions, and ultimately generates small molecule phenolic acids, phenylpropionic acid derivatives; these phenolic intermediates can further be metabolized into short-chain fatty acids (SCFA) such as acetic acid and butyric acid [24,25]. Compared to the prototype substrate, the molecular weight of the III phase metabolites is significantly reduced, the intestinal permeability is stronger, and the trans-organ transport efficiency is higher, thus becoming a core functional medium with both signal regulation potential and targeted physiological activity [26].

The distinctive feature that distinguishes honey from single-plant-source phenolic supplements lies in its inherent ability to simultaneously deliver “oligosaccharides + phenols” to the intestinal tract, thereby generating a dual synergistic effect [10]. For instance, the naturally occurring oligosaccharides in buckwheat honey, such as kestose, isomaltose, isomalt triose, and panose, can selectively enrich core phenolic metabolic bacteria like *Bifidobacterium*, thereby enhancing the overall metabolic enzyme capacity of the body for phenolic substrates [11]. The anaerobic fermentation of oligosaccharides in the intestinal tract not only promotes the massive production of short-chain fatty acids but also creates favorable conditions for the microbial transformation of phenolic compounds by reshaping the bacterial community structure. Research using the SHIME^®^ in vitro intestinal model has confirmed that the mixed low-fiber sugar and polyphenols are fully fermented throughout the colon, increasing butyric acid production in the ascending colon by 7 mmol/L, and increasing acetic acid, propionic acid, and butyric acid by approximately 4 mmol/L in the transverse and descending colon, while simultaneously increasing the abundance of *Lactobacillus* and *Bifidobacterium* by approximately 2.5 Log/mL [27]. Animal experiments demonstrated that adding fructo-oligosaccharides (FOS) together with polyphenol concentrate (PC) rich in quercetin glycosides to the feed enhanced the adaptive response of the cecal microbial enzymatic system, as evidenced by the increased susceptibility of rutin, hyperoside, and quercitrin to hydrolysis by cecal contents [28]. This suggests that stimulation of the intestinal microbiota by prebiotics may enhance the body’s metabolism of dietary phenolic compounds. In buckwheat honey, the naturally coexisting oligosaccharides (kestose, isomaltose, isomalt triose, and panose) and 12 phenolic compounds (catechin, gallic acid, p-coumaric acid, etc.) can be simultaneously delivered to the intestinal tract. In vitro fermentation experiments have confirmed that they can selectively promote the growth of beneficial bacteria like *Bifidobacterium* and inhibit pathogenic bacteria [10]. This evidence indicates that oligosaccharides and phenols in honey function in the intestinal tract through a coordinated metabolic interaction: oligosaccharides serve as fermentable carbon sources that selectively promote the growth of phenolic-metabolizing bacteria, while phenolic substrates are simultaneously transformed by the microbiota. This coordinated utilization of both substrates creates a synergistic microenvironment that enhances the overall metabolic capacity of the gut microbiota.

### 3.3. From Phenolic Precursors to Bioactive Microbial Metabolites

Gut microbial metabolism is the core link for honey phenolic compounds to exert their health effects [29]. Current evidence indicates that the endogenous phenolic components act primarily as precursor substrates, while the microbial-derived small-molecule metabolites are likely the major mediators of systemic effects [30,31]. These metabolite molecules have an appropriate molecular weight and balanced lipid solubility and can efficiently penetrate the intestinal mucosal barrier. Some monomers can even cross the blood–brain barrier, achieving multi-target regulation throughout the body [32]. Studies have shown that the metabolite (3,4-DHPAA) of quercetin produced by the microbiota can penetrate the intestinal barrier and enter the systemic circulation. In vitro studies have demonstrated that 3,4-DHPAA can significantly inhibit the secretion of pro-inflammatory cytokines by human peripheral blood mononuclear cells stimulated by LPS [7]. This discovery reinforces the cognitive framework of “phenols as precursors and metabolites as effect molecules”.

## 4. Honey Phenolic Substances’ Intestinal Microbiota Metabolic Pathway

### 4.1. Metabolic “Three-Step Process”

**Figure 1:**
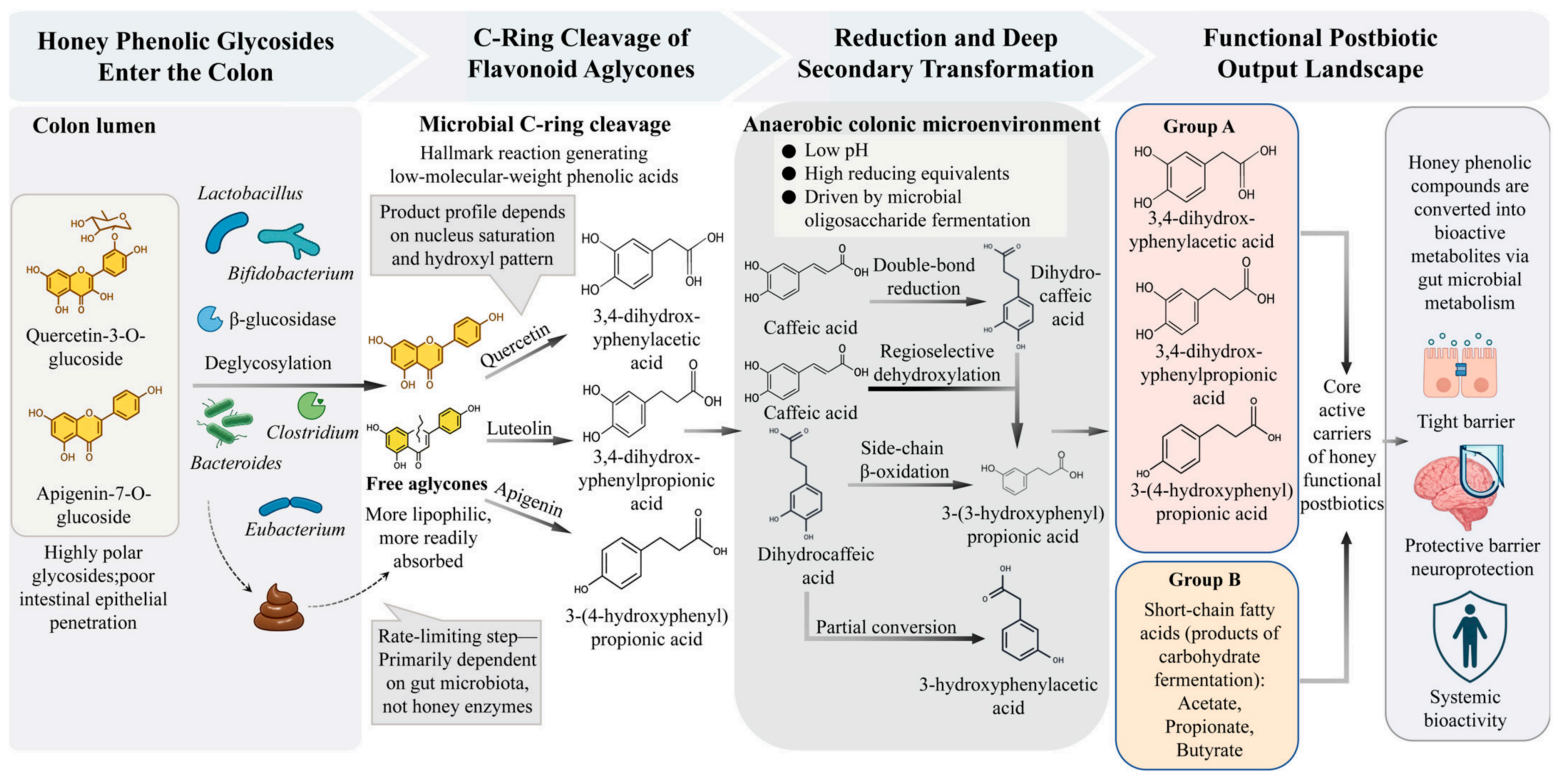
Three-step metabolic pathway of honey phenolic glycosides under the action of colonic microbiota.

The transformation of honey phenolic substances by the intestinal microbiota is a highly specific, enzymatically controllable, cascading biochemical process, rather than random degradation [33]. In the colonic microecology, honey-derived phenols follow a fixed sequential metabolic pathway of “deglycosylation–C-ring cleavage–reduction and deep secondary transformation”, gradually completing structural modification and molecular activation (Figure 1). This process is driven primarily by the functional enzyme system of gut microbiota. The close linkage between upstream and downstream metabolites governs the degradation rate of phenolic substrates and the bioactivity of the resulting metabolites [6].

In the colonic environment, deglycosylation is the core rate-limiting step of the entire metabolic pathway. Natural honey phenols mostly exist in glycoside-bound forms, such as quercetin-3-O-glucoside and apigenin-7-O-glucoside [6,34]. Due to their high polarity, these glycosides are difficult to penetrate the intestinal epithelial barrier and must first dissociate the sugar side chains to release liposoluble and more easily absorbed free glycosides [35,36]. The efficiency of deglycosylation directly determines the subsequent metabolic process. If this step is blocked, the intact phenolic glycoside precursors reaching the colon will be excreted with feces [23]. Therefore, the background activity of glycosidases in individual intestinal microbiota is the primary bottleneck determining the metabolic direction of honey phenols in the colon. Although honey itself contains α-glucosidase and β-glucosidase, these endogenous enzymes have natural substrates mainly of low-molecular-weight oligosaccharides and disaccharides, and lack efficient and specific hydrolytic activity for phenolic glycosides. Therefore, this rate-limiting step in the colonic stage is mainly completed independently by the symbiotic microbiota in the intestine [6,37].

C-ring-specific cleavage is the most distinctive and significant core reaction in microbial metabolism, targeting the oxygen-containing heterocyclic framework of the flavonoid parent nucleus and generating low-molecular-weight, low-polarity phenolic acid derivatives. The classical pathway is quite clear: quercetin is metabolized into 3,4-DHPAA, luteolin is degraded into 3,4-DHPPA, and apigenin undergoes reduction and ring-opening to form 3-(4-hydroxyphenyl) propionic acid [25]. The saturation degree of the parent nucleus, as well as the number and location of hydroxyl groups of the phenolic substrate, directly determine the differentiation of the cleavage product profile [24]. After flavonoids are metabolized by the intestinal microbiota, they undergo ring-opening reactions to form low-molecular-weight phenolic acid precursors. These small-molecule metabolites not only optimize lipid solubility but have also been proven to possess significant multi-target biological activities such as neuroprotective effects, thereby significantly expanding the regulatory coverage of the metabolites [38].

Reduction and deep secondary transformation are the final key steps in reshaping the active configuration of phenolic compounds. The small-molecule phenolic acids generated by the cleavage of the C-ring can undergo further deep modifications such as side-chain double bond reduction and aromatic ring directional dehydroxylation in the anaerobic environment created by the microbiota. At the same time, the fermentation of oligosaccharides by the microbiota will locally construct a microenvironment with a low pH and high reduction equivalents, providing essential cofactors for a series of reduction reactions [6]. Representative reactions include: the reduction in the side chain double bond of caffeic acid to dihydrocaffeic acid, and the dehydroxylation of dihydrocaffeic acid to form 3-(3-hydroxyphenyl)propionic acid; some products can also be converted through side-chain β-oxidation into downstream metabolites such as 3-hydroxybenzoic acid [39]. The series of aromatic derivatives thus generated, together with the short-chain fatty acids (acetic acid, propionic acid, butyric acid) produced by parallel fermentation of honey carbohydrate components, jointly constitute the core active carrier of honey functional precursors [11]. This closed-loop three-step metabolic pathway works in synergy with the parallel fermentation chain of carbohydrates, constructing a complete intestinal metabolic map for the efficient conversion of dietary phenols into microbial metabolites.

### 4.2. Coupling of Key Enzymatic Adaptation Characteristics of Core Metabolic Functional Bacterial Genera

The global metabolic process of honey phenolic compounds is accomplished through the collaborative symbiosis of multiple bacterial strains. A single bacterial strain cannot independently complete the three-step interconnected pathway. Different dominant bacterial genera perform orderly division of labor and functional complementarity at each metabolic node. The β-glucosidase-producing bacterial genus spectrum is broad, covering *Lactobacillus*, *Bifidobacterium*, and *Bacteroides* genera [40,41]. The substrate targeting preference of the strains shows a trend of differentiation. Preliminary studies have shown that some *Bifidobacterium* species can effectively degrade flavonoid-7-O-glucoside, while some *Lactobacillus* species exhibit hydrolytic activity towards flavonoid-3-O-glucoside [40]. The *Bacteroides* genus, as the dominant bacterial community in the intestine, has a genome encoding a rich repertoire of glycoside hydrolases, which has been proven to play a key role in de-oligoglycosylation reactions. The range of adaptability of the rhamnosidase-producing strains is narrow; stable expression is mainly found in *Lactobacillus* and a few *Clostridium* strains. The bacterial genus spectrum with exclusive functions for the cleavage of the C ring of flavonoids is highly specific, including the genera *Clostridium*, *Eubacterium*, *Eggerthella*, and *Flavonifractor plautii* [38]. The substrate structure recognition specificity of the strains is extremely strong: *F. plautii* efficiently cleaves unsaturated parent nucleus flavonol substrates such as quercetin and kaempferol; some strains of the *Clostridium* genus have cleavage activity for flavonoids such as luteolin; the *Eubacterium* genus has outstanding cleavage efficacy for saturated parent nucleus flavanone substrates; the *Eggerthella* genus mainly carries the dehydroxylation function enzyme system of aromatic rings (such as the *E. lenta* CAT-1 strain cleaves the C ring of catechin and then continues to complete the B ring dehydroxylation) [38]. The abundance of the above functional bacteria in the intestinal tracts of healthy individuals is naturally heterogeneous. This individual-level variation in the composition and function of the intestinal microbiota is precisely the core endogenous trigger for the differential metabolic phenotypes of honey phenolic compounds in different individuals [42,43].

The endogenous oligosaccharide components of honey can exert precise prebiotic regulatory effects, directing the enrichment of key glycoside hydrolase-producing bacteria. The indigestible low oligosaccharides such as pectinose, pectinose-2, and fructo-oligosaccharide directly reach the colon. The specifically proliferating *Bifidobacterium*, *Lactobacillus*, and *Faecalibacterium* genera simultaneously enhance the biomass of the microbiota and the overall glycoside enzyme reserve capacity. The enriched microbiota ecological niche provides a favorable microenvironment for the collaborative metabolism of C ring cleavage bacteria, jointly constituting the microecological foundation of the “sugar-phenol collaborative metabolism” of honey [9,12].

Beyond the functional division at the genus level, the enzymatic adaptability and ecological mechanisms underlying honey phenolic metabolism warrant further exploration. In the deglycosylation stage, different bacterial genera encode glycoside hydrolase (GH) families with distinct substrate preferences: *Bifidobacterium* strains predominantly express GH3 family β-glucosidases that display preferential activity toward flavonoid 7-O-glucosides, whereas certain *Lactobacillus* species deploy GH1 family enzymes with higher affinity for flavonoid 3-O-glucosides. Such substrate differentiation facilitates niche partitioning among bacterial taxa and improves overall metabolic performance of gut microbial communities [44,45]. Concurrently, widespread β-glucosidase activity across multiple genera establishes functional redundancy; suppression of one taxon can be compensated by others to sustain continuous bioconversion of phenolic substrates [44]. In the C ring cleavage step, *Flavonifractor plautii* shows stringent substrate selectivity for unsaturated flavonols such as quercetin, requiring prior reduction to saturate the C ring double bond before ring opening. By contrast, *Eubacterium ramulus* possesses both reductive and ring-cleavage activities and efficiently transforms quercetin into 3,4-DHPAA [6,40]. The naturally coexisting “oligosaccharide–phenolic” matrix in honey may enhance enzymatic adaptability via synergistic induction. Short-chain fatty acids generated during oligosaccharide fermentation lower local pH and supply reducing equivalents, generating favorable microenvironmental conditions for C ring cleavage and subsequent reduction reactions [5,12].

### 4.3. Inter-Species Cross-Feeding Interactions and Phenolic Closed-Loop Metabolic Network

The honey phenolic intestinal metabolism is not an isolated linear biochemical reaction, but a complex micro-ecological metabolic network formed by substrate mutual feedback and metabolite sharing among multiple species. The inter-species cross-feeding effect is the core link maintaining the stable operation of the network. Taking quercetin as an example, *Clostridium perfringens* and *Bacteroides fragilis*, among other core functional bacteria, can degrade it into 3,4-DHPAA [46]. In terms of inter-species cross-feeding, a study using *Eubacterium ramulus* as a model confirmed that although *E. ramulus* independently degrades quercetin, co-culture with *Bacteroides thetaiotaomicron* significantly enhances this degradation activity. Mechanistically, *B. thetaiotaomicron* hydrolyzes starch to provide fermentable carbon sources for *E. ramulus*, thereby supporting its growth and metabolic activity. This cross-feeding effect suggests that the availability of carbon sources is a key ecological factor modulating the efficiency of flavonoid metabolism [47]. Based on this, it can be reasonably assumed that the 3,4-DHPAA generated by the core strain may continue to be the substrate for downstream symbiotic bacteria without C-ring cleavage ability, undergoing deep degradation to generate various phenolic acid products such as 3,4-dihydroxybenzoic acid (3,4-DHBA) and 3,4,5-trihydroxybenzoic acid (3,4,5-THBA), achieving a relay metabolic transformation. Sankaranarayanan’s research found that in the screening of quercetin-degrading bacteria, phenolic acid products such as 3,4-DHBA were detected in a single-strain culture system, but this type of research system is difficult to strictly distinguish whether the metabolites are independently completed by a single strain (parallel metabolism) or require successive actions of different strains (relay metabolism) [48]. The exact indirect metabolic pathway of the species still needs to be verified through multi-strain co-culture and metabolite tracking experiments.

Based on this inter-species cross-feeding, the phenolic closed-loop metabolic network is also subject to feedback regulation by metabolites. The fermentation of sugars in honey can regulate phenolic metabolism from multiple dimensions. The butyric acid-producing bacteria themselves are involved in the C-ring cleavage of flavonoids, suggesting functional coupling [49]. In addition, SCFA, as classic histone deacetylase (HDAC) inhibitors, have been proven to regulate host gene expression through HDAC inhibition, providing theoretical support for the hypothesis that SCFA may further upregulate the expression of related microbial metabolic enzyme genes, but this direction still requires direct experimental verification [50]. The weak acidic environment makes β-glucosidase and rhamnosidase approach the active pH range [51,52]; at the same time, SCFA fermentation maintains the supply of NADH/NADPH cofactors, supporting the reduction step [53]. This interaction is a network with both positive feedback and redundancy, and should not be simplified to a unidirectional closed loop.

### 4.4. Differences in the Regulation of Metabolite Profiles by Honey Source Genotypes and Maturation Degree

The genetic differences in honey sources directly determine the basic phenolic fingerprint pattern of honey, thereby inducing differential metabolic phenotypes in the microbial community. The characteristic phenols of Manuka honey are mainly composed of lignans such as pinoresinol, while buckwheat honey is rich in rutin and quercetin, which are unsaturated flavonols. Previous studies have shown that the flavonoid lyase (Fcr) in the intestinal microbiota has strict substrate specificity and can specifically recognize and cleave flavonoids with saturated C-ring structures, converting them into dihydrochalcones, which can be further metabolized by the intestinal microbiota into 3-phenylpropionic acid derivatives, but cannot act on unsaturated flavonols [54]. Based on this, it can be reasonably speculated that saturated flavonoids, under the action of the microbiota, are expected to differentiate towards 3-phenylpropionic acid derivatives, possibly focusing on repairing the intestinal mucosal barrier and regulating the neural activity of the gut–brain axis; while unsaturated flavonols, due to their inability to be cleaved by Fcr, will be metabolized through other microbial pathways and produce dihydroxybenzene acid derivatives, targeting to mediate the anti-inflammatory homeostasis of the body [55]. However, the above metabolic pathways and their functional attributions still need to be verified through strain fermentation and structural identification experiments. The heterogeneity of the prebiotic functions of honey sources is essentially an external manifestation of the differences in phenolic substrates after microbial metabolism, which can provide a core theoretical support for the precise functional classification of honey.

The maturation degree of honey is also a key regulatory variable that is often overlooked. Natural mature honey has low moisture content and high endogenous enzyme activity, and has not undergone high-temperature artificial processing; non-mature honey is treated by heat concentration technology, and the endogenous functional enzymes have significantly declined, accompanied by the formation of advanced glycation end products (AGEs) through the Maillard reaction [56]. Previous studies have shown that glucose oxidase can indirectly optimize the metabolic environment of the microbiota by regulating the intestinal redox potential; while AGEs can inhibit the colonization of core beneficial metabolizing bacteria—in vitro fermentation shows that activity of advanced glycation end products (AGEs) can reduce the abundance of *Bacteroidetes* by more than 10 times within 8 h, and the abundance of core beneficial bacteria such as *Bifidobacterium* and *Lactobacillus* decreases, while opportunistic pathogenic bacteria proliferate [57,58].

Beyond the antibacterial effects of AGEs, industrial processing temperatures can damage the physicochemical structure of honey. Thermal treatment (especially exceeding 50–60 °C) not only significantly reduces the activity of endogenous β-glucosidase and glucose oxidase, but also accelerates the Maillard reaction to generate substantial hydroxymethylfurfural (HMF), while concurrently consuming prebiotic-active oligosaccharides and altering the ratio of glycosidically bound to free phenolic compounds [59]. Thereby potentially undermining the microbial ecological foundation of the “sugar-phenol synergistic metabolism. Therefore, the maturation degree of honey can indirectly intervene in the overall efficacy of phenolic microbial community metabolism, and it is urgently needed to be included in the subsequent mechanism verification and population intervention research system. To visually illustrate how different floral genotypes direct characteristic microbial metabolites through structural differences in their phenolic constituents, Table 1 summarizes the key phenolic components, structural–metabolic determinants, and corresponding microbial metabolic transformation pathways for representative honeys.

## 5. Regulation of the Bioavailability of Honey Phenolic Substances by Gut Microbiota Metabolism

Significant differences exist in the characteristic phenolic compounds of honeys from different botanical origins. These differences directly influence the gut microbiota metabolic profiles and potential health effects. For systematic comparison, the phenolic compositions, major microbial metabolites, key microbiota shifts, and corresponding health benefits of representative honey sources are summarized in Table 2.

### 5.1. Definition of Bioavailability: From Systemic Exposure to Local Exposure

In traditional pharmacokinetics, when evaluating the bioavailability of orally administered substances, the plasma concentration of the drug in its original form is usually the core indicator, including peak concentration (Cmax), area under the plasma concentration-time curve (AUC), and time to peak concentration (Tmax), etc. [69]. However, when this classic framework is applied to honey phenolic substances, a significant “underestimation” problem emerges. As mentioned earlier, the concentration of phenolic substances in their original form in the plasma after oral administration is only at the nmol/L level, far below the in vitro effective concentration. This does not mean that these phenolic substances have no biological activity in the body; it suggests that the traditional evaluation system based on systemic exposure may not be applicable to such natural products that highly rely on microbiota metabolism.

To address this contradiction, researchers proposed the “local high concentration in the intestine” hypothesis. The core idea of this hypothesis is that phenolic substances and their metabolites produced by the microbiota can reach much higher concentration levels in the intestinal lumen and intestinal mucosa, typically reaching the μmol/L or even mmol/L level [24]. The formation of this local high concentration is based on two reasons. First, honey phenolic substances enter the intestine at a higher dose, concentrating in the intestinal contents before being absorbed, resulting in naturally higher local concentrations. Second, the phenolic metabolites form concentration gradients on the intestinal mucosa surface and directly contact the epithelial cells. Although the proportion that enters the circulation system is limited, these molecules are sufficient to activate multiple signaling pathways in the local area. Therefore, when evaluating the bioavailability of honey phenolic substances, “local exposure in the intestine” should be taken into consideration, rather than merely focusing on the original plasma concentration. This redefinition helps explain the apparent contradiction of “low plasma drug concentration but physiological effect”.

### 5.2. Three Pathways for Improving Absorption Through Microbial Metabolism

The third-phase metabolism of the intestinal microbiota synergistically enhances absorption from three dimensions: physical-chemical structure, molecular size, and the intestinal barrier microenvironment. This process systematically overcomes the absorption bottleneck of honey phenolic compounds and promotes the formation of bioactive metabolites.

The first pathway is the enhancement of lipid solubility due to de-sulfonation. As mentioned earlier, most honey phenolic compounds exist in the form of glycosides, and the presence of the sugar group significantly increases the polarity of the molecule, making it difficult for passive diffusion to pass through the lipid bilayer of intestinal epithelial cells. The aglycones released after the removal of the sugar group by β-glucosidase and rhamnosidase have significantly enhanced lipid solubility, with an increase in the oil-water partition coefficient (log P), and thus possess the ability for passive transmembrane transport [38]. This is the most direct mechanism for improving absorption through microbial metabolism.

The second pathway is the reduction in molecular weight due to C-ring cleavage. The molecular weight of flavonoid aglycones is typically between 250 and 300 Da. Although they already have some transmembrane ability, they are still limited by the molecular volume. After C-ring cleavage, the small molecule phenolic acid metabolites generated have a molecular weight typically below 200 Da, and some even below 150 Da. These small molecules can not only be absorbed through the transcellular pathway but also passively diffuse through the paracellular pathway between intestinal epithelial cells, that is, through the tight junction gap, bypassing the limitation of transmembrane transport, and further improving the absorption efficiency [3].

The third pathway is the optimization of intestinal barrier function mediated by SCFA. The large amount of SCFA produced by the fermentation of honey sugars, especially butyric acid, is not only the preferred energy substrate for intestinal epithelial cells but also activates the AMPK signaling pathway to promote the reorganization and expression of tight junction proteins (such as Claudin-1, Occludin, ZO-1), enhancing the integrity of the intestinal barrier [70]. The effect of butyric acid in reducing intestinal permeability can reduce the translocation of endotoxin across the barrier, thereby alleviating the low-level inflammatory drive of metabolic endotoxemia to distant organs (such as the liver, adipose tissue) [71]. Therefore, the regulatory effect of SCFA on intestinal permeability mainly lies in maintaining barrier integrity and reducing systemic inflammation, rather than directly increasing the paracellular absorption of phenolic metabolites.

### 5.3. Intestinal-Liver Circulation of Metabolites and Secondary Exposure

The fate of phenolic substances in honey does not end with a single absorption. The phenolic metabolites that are absorbed into the circulatory system, whether they are the original aglycones or small molecules produced by the microbiota metabolism, undergo a second combination reaction in the liver—glucuronidation or sulfation—after which their polarity significantly increases, facilitating their excretion back into the intestinal tract via bile. This process itself is a common way for the body to clear exogenous substances, but for phenolic metabolites, bile excretion does not mean the end of the metabolic pathway [72]. The ubiquitous β-glucuronidase (GUS) in the intestinal microbiota can hydrolyze the glucuronide conjugates, reactivating the metabolites to their free form, which can be reabsorbed into the circulatory system again, forming an intestinal-liver circulation [73]. This reactivation-reabsorption process prolongs the residence time of phenolic metabolites in the body, allowing their effects to last much longer than expected from a single absorption. Additionally, certain phenolic components in honey can themselves regulate the activity of the β-glucuronidase of the microbiota. For example, quercetin and its microbiota metabolites have been reported to inhibit the activity of this enzyme, thereby slowing the rate of dissociation of the bound metabolites [74]. The result of this inhibitory effect is that the metabolites remain in the intestinal tract for a longer time in the bound form, although the bound form itself has no direct activity, but its persistent presence provides a more lasting substrate pool for the gradual dissociation of the microbiota, thereby prolonging the exposure time of free active metabolites in the local intestinal area and enhancing the local effect rather than the systemic effect. This “self-prolongation” regulatory mechanism reflects the complex feedback relationship between honey phenolic substances and their microbiota metabolism.

### 5.4. Individual Variability in Bioavailability: The Dominant Role of Gut Microbiota

**Figure 2:**
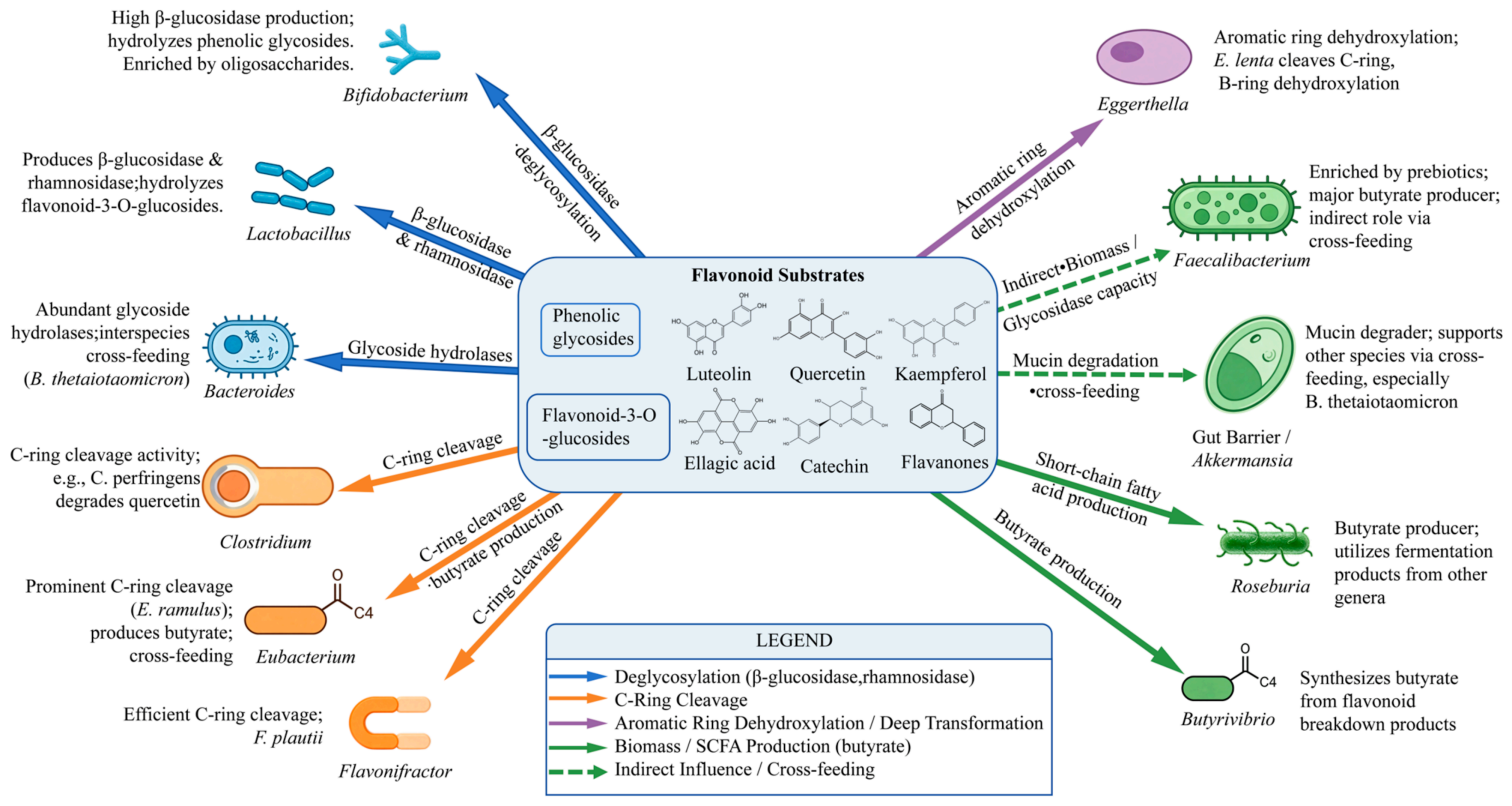
Core gut bacterial genera, key enzyme systems, and physiological functions that participate in the metabolism of honey phenolic compounds.

There is a significant individual variation in the bioavailability of the same phenolic compounds among different individuals, which has become a consensus in this field. The factors contributing to this variation are numerous. For instance, the specific types and diversity of dietary polyphenols can positively correlate with the abundance of gut microbial genes encoding phenolic-utilizing pathways (PUP) and related bacterial genera, suggesting that diet may help shape the gut’s phenolic-transforming potential [75]. Aging significantly impairs this metabolic capacity; for instance, taking ferulic acid-a signature phenolic acid in cereals-as an example, human fecal fermentation combined with multi-omics analyses revealed that while the gut microbiota from individuals across all age groups could degrade ferulic acid, the process exhibited substantial inter-individual variability, with significantly reduced degradation efficiency and conversion rates observed in the elderly population [76]. Animal experiments showed that in mice treated with antibiotics to deplete the gut microbiota and fed a tea polyphenol diet, significant accumulation of the intact catechin (–)-epigallocatechin-3-gallate (EGCG) was observed in serum and liver, and multiple intact catechins, including EGCG and (–)-epicatechin-3-gallate (ECG), were detected in urine [77]. Moreover, clinical cohort studies have highlighted the disorder of polyphenol–microbiota metabolism under pathological conditions; compared with healthy controls, patients with Crohn’s disease not only consumed lower levels of dietary polyphenols but also exhibited significantly impaired flavonoid-degrading capacity of their gut microbiota, alongside markedly reduced serum levels of hippuric acid, a signature metabolite of polyphenol metabolism [78]. Notably, beyond these modulatory factors, an increasing body of evidence indicates that the baseline compositional structure of the gut microbiota-namely, the enterotype-is the most important determining factor [72]. The human gut microbiota can be roughly divided into three main enterotypes: *Bacteroides*-dominated, *Prevotella*-dominated, and *Ruminococcus*-dominated. These three enterotypes show systematic differences in the gene abundance encoding carbohydrate-active enzymes (CAZyme), glycoside hydrolases, and phenolic metabolism-related enzymes (Figure 2) [79,80].

Studies have found that in the fecal fermentation systems of individuals with different enterotypes, there are significant differences in the metabolic product spectrum and metabolic rate. The gut microbiota of individuals with the *Prevotella*-dominated type tends to efficiently convert ellagic acid into a single final product, “urolithin A”, while the microbiota of the *Bacteroides*-dominated type will further convert urolithin A into a more complex mixture of metabolites such as “urolithin B”, resulting in the accumulation or depletion of intermediate products (such as urolithin A), presenting a completely different in vivo exposure profile [31]. This enterotype-dependent metabolic difference directly affects the final exposure level and active metabolite profile of phenolic compounds in the body.

The most direct causal evidence comes from animal models pre-treated with antibiotics. Studies have shown that mice with their gut microbiota cleared by broad-spectrum antibiotics, when given phenolic extracts by gavage, have significantly lower plasma concentrations of phenolic compounds than normal mice [81]. This sharp decrease cannot be explained by other changes in absorption or metabolic pathways; the only variable is the absence of the microbiota. This result strongly proves that the gut microbiota is a critical determinant of bioavailability for many honey phenolic compounds, and its presence substantially enhances absorption and metabolic conversion for the majority of these substrates. Without the participation of the microbiota, the absorption of phenolic compounds is almost impossible to occur effectively. This discovery has important clinical implications for understanding the phenomenon of the decline in the efficacy of dietary supplements during antibiotic use and provides a theoretical basis for the concept of “precision honey nutrition”—that is, personalized recommendations for honey types and doses based on individual enterotype characteristics to optimize the output efficiency of prebiotics.

## 6. Multi-Target Regulation of Host Health by Gut Microbial Metabolites

### 6.1. Gut–Liver Axis: Reducing Metabolic Endotoxemia and Non-Alcoholic Fatty Liver Disease

**Figure 3:**
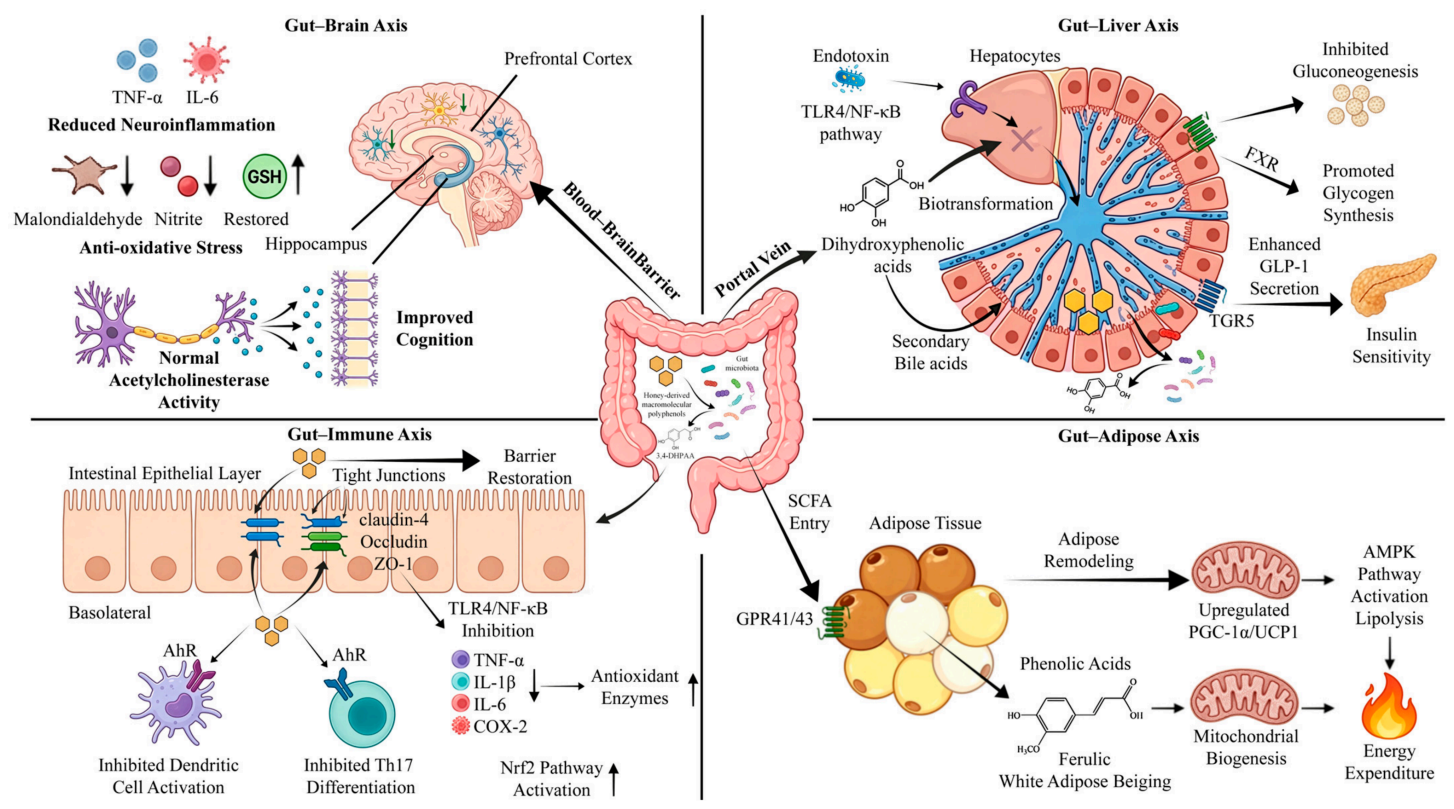
Multi-target regulatory mechanisms of honey-derived gut microbiota metabolites on host health via the four major gut axes. ↑ indicates increase (e.g., elevated biomarkers, activated pathways, enhanced functions, increased expression); ↓ indicates decrease (e.g., reduced biomarkers, inhibited pathways/activities, weakened processes).

Honey is rich in various polyphenolic compounds such as flavonoids and phenolic acids. These macromolecular polyphenols, due to their low bioavailability, mostly do not get absorbed by the small intestine but enter the colon, where they are metabolized by the gut microbiota into small-molecule phenolic acids, such as 3,4-DHPAA, which are typical phenolic metabolites derived from gut microbiota metabolism [7]. These metabolites, after entering the liver *via* the portal vein, do not merely act locally in the intestine but exert systemic liver-protective functions through the gut–liver axis (Figure 3).

At the molecular mechanism level, 3,4-dihydroxyphenolic acids can inhibit the Toll-like receptor 4 (TLR4)-mediated nuclear factor-κB (NF-κB) inflammatory signaling pathway. TLR4 is the key pattern recognition receptor for recognizing intestinal-derived endotoxin LPS—a high-fat diet can disrupt the integrity of the intestinal barrier, allowing LPS to enter the liver via the portal vein, continuously activating TLR4 and triggering metabolic endotoxemia and liver inflammation. Monagas et al. confirmed through in vitro experiments that 3,4-DHPPA and 3,4-DHPAA produced by gut microbiota metabolism can significantly inhibit LPS-stimulated pro-inflammatory cytokine secretion in human peripheral blood mononuclear cells: the secretion inhibition rate of TNF-α reached 84.9% and 86.4%, respectively, and the level of IL-6 decreased by 88.8% and 92.3% [7]. This indicates that honey phenolic metabolites can inhibit the inflammatory response mediated by NF-κB and effectively alleviate liver inflammation burden [82].

At the same time, these phenolic metabolites also participate in regulating bile acid metabolism and promoting the generation of secondary bile acids. Liu et al. pointed out that dietary polyphenols can regulate bile acid metabolism through two modes: one is directly activating/inhibiting farnesoid X receptor (FXR) and Takeda G protein-coupled receptor 5 (TGR5); the other is indirectly influencing bile acid synthesis and metabolism by changing the composition of gut microbiota [83]. Secondary bile acids act as endogenous ligands for FXR and TGR5. Activation of FXR can inhibit gluconeogenesis and promote hepatic glycogen synthesis, while activation of TGR5 stimulates the secretion of glucagon-like peptide-1 (GLP-1), enhancing insulin sensitivity [83]. Abdul Ghani et al.’s research provided direct evidence of honey’s liver-protective effect. In a rat model of non-alcoholic fatty liver disease (NASH) induced by high cholesterol diet, Tualang honey intervention (1.2 g/kg/day) significantly reduced serum ALT (77.25 → 50.25 U/L, *p* = 0.025) and AST (193.25 → 106.75 U/L, *p* < 0.01), and significantly improved fasting insulin, insulin resistance index (HOMA-IR), and lipid profile; histological analysis of liver tissue confirmed that the NAFLD activity score (NAS) was significantly improved in the treatment group [84]. Therefore, the phenolic metabolites generated by the transformation of honey phenolic compounds by gut microbiota exert multi-target protection against non-alcoholic fatty liver disease via the gut–liver axis, by both inhibiting the TLR4/NF-κB inflammatory pathway to alleviate metabolic endotoxemia and regulating bile acid metabolism through the FXR/TGR5 axis to improve hepatic glycolipid metabolism disorders.

### 6.2. Gut–Brain Axis: Anti-Neuroinflammation and Cognitive Protection

These small-molecule phenolic metabolites have low molecular weights and moderate lipid solubility, which facilitate their potential to penetrate the blood–brain barrier. With higher bioavailability than their parent compounds, they can partially cross the blood–brain barrier to enter the central nervous system, thereby exerting remote regulatory effects through the gut–brain axis [8,85]. The phenolic acid metabolites that enter the brain can inhibit neuroinflammation through multiple target mechanisms: on the one hand, they significantly reduce the levels of pro-inflammatory factors TNF-α and IL-6, and alleviate the excessive activation of microglia; on the other hand, they effectively reduce the content of malondialdehyde and nitrite in the brain, reverse the depletion of reduced glutathione, and alleviate oxidative stress damage [86]. At the cognitive protection level, these metabolites can restore the abnormal increase in acetylcholinesterase activity in key brain regions such as the hippocampus and prefrontal cortex, and promote the restoration of neuronal arrangement order and the normal distribution of Nissl bodies at the histological level, thereby effectively improving memory impairment and cognitive decline caused by neuroinflammation [86].

### 6.3. Gut–Immune Axis: Systemic Immune Regulation

The phenolic compounds in honey (such as quercetin, apigenin, caffeic acid, etc.) are metabolized by the gut microbiota to generate low-molecular-weight phenolic metabolites, such as 3,4-DHPAA, uric acid A, and gallic acid, which achieve systemic immune regulation through the gut–immune axis [87,88]. Firstly, these metabolites can upregulate the expression of intestinal epithelial tight junction proteins (claudin-4, occludin, ZO-1), repair the intestinal barrier function, and reduce the diffusion of bacterial products to the circulatory system, thereby inhibiting the triggering of systemic low-grade inflammation [87,89]. Secondly, phenolic metabolites act as agonists of the aryl hydrocarbon receptor (AhR) and can regulate local immune responses in the intestine; for example, uric acid A inhibits the activation of dendritic cells and the differentiation of Th17 cells through the AhR-dependent pathway [87]. In addition, honey and its metabolites can significantly inhibit the TLR4/NF-κB signaling pathway, systemically downregulate the expression of pro-inflammatory cytokines such as TNF-α, IL-1β, IL-6, and COX-2, and some metabolites (such as catechol, uric acid A) can activate the Nrf2 pathway to enhance antioxidant defense, reducing the damage of oxidative stress to immune cells [87,88,89]. It is worth noting that the composition of phenolic compounds in honey from different sources and different bee species varies significantly, resulting in dose-dependent and microbiota-dependent immune regulatory effects, providing a theoretical basis for the development of personalized immune-regulating functional foods [87,88,89].

### 6.4. Gut–Fat Axis: Brownification of White Fat and Energy Homeostasis

The phenolic compounds in honey, as natural prebiotics, can be metabolized to generate various microbial metabolites, including SCFA and phenolic derivatives [67,90]. These metabolites mediate the brownification of white fat and the regulation of energy homeostasis through the bidirectional communication network of the gut–fat axis [91]. Specifically, honey polyphenols and their metabolites can selectively enrich beneficial gut bacteria, such as *Lactobacillus* and *Akkermansia*, and promote the production of short-chain fatty acids like acetic acid and propionic acid [90]. SCFA can activate the GPR41/GPR43 receptors in adipose tissue, thereby initiating downstream thermogenic programs and enhancing the activity of brown adipose tissue and the beigeification of white fat [67,91]. Additionally, phenolic acids such as vanillic acid and ferulic acid in honey, along with their microbial metabolites, can directly act on white adipose tissue, upregulate the expression of PGC-1α and UCP1, induce the formation of beige fat cells, and enhance mitochondrial biogenesis [67,92]. At the same time, these active components can activate the AMPK signaling pathway, promote fat breakdown and energy consumption, and improve intestinal barrier function and alleviate chronic low-grade inflammation [67,90,91]. It is worth noting that the effects of honey from different nectar sources in regulating energy homeostasis through the gut–fat axis vary significantly due to differences in phenolic composition. Among them, honeys rich in polyphenols, such as those from poplar trees, exhibit more prominent anti-obesity and metabolic improvement effects [90].

### 6.5. Quantitative and Temporal Characteristics of Microbial Metabolites

The microbial metabolites (such as 3,4-dihydroxybenzoic acid, protocatechuic acid, methyl eugenol, etc.) produced by the metabolism of honey phenols by the intestinal microbiota have a significant dose-dependent regulatory effect on the host’s health, and there are significant differences in the effective physiological concentration ranges of different metabolites. In vitro studies have shown that phenolic acids derived from microorganisms and their complexes can exert neuroprotective and anti-inflammatory effects within the physiological concentration range of 1 to 50 μM; among them, 3,4-dihydroxybenzoic acid and dihydrocaffeic acid can significantly inhibit the secretion of TNF-α and IL-6 in macrophages stimulated by LPS, while their glucuronide conjugate (DHCFAg) exhibits stronger biological activity [93]. In animal experiments, the main intestinal metabolite of flavonoids in honey, protocatechuic acid, was administered at a daily dose of 5 mg/kg and 25 mg/kg for 15 days, which could dose-dependently alleviate intestinal damage in mice with DSS-induced colitis and significantly restore the membrane distribution of ZO-1 and claudin-2/4 in the colon tissue. Among them, the 25 mg/kg dose group showed better repair effect on the distribution of tight junction proteins than the 5 mg/kg group, and could more effectively reduce the mRNA expression levels of IL-1β and TNF-α in the colon tissue [94]. In addition, the characteristic metabolite of manuka honey, methyl syringate, can dose-dependently inhibit the release of inflammatory mediators from neutrophils within the concentration range of 600 μM to 1300 μM, and at 1300 μM, it can reduce the release of myeloperoxidase by about 70% [62].

The biological effects of honey phenolic microbial metabolites also have a clear temporal dependency. At the cellular level, the inhibitory effect of manuka honey and its metabolite methyl syringate on the release of inflammatory mediators from neutrophils increases with time: treatment for 3 h can significantly reduce the level of myeloperoxidase (MPO), and the inhibitory effect on IL-8 reaches its strongest at 6 h, reducing its level to nearly baseline [62]. This time-dependent effect suggests that when applying such microbial metabolites, their dynamic change characteristics need to be considered to optimize the administration strategy.

### 6.6. Summary of Evidence from In Vitro, Animal, and Clinical Studies

To provide a structured overview of the current evidence base, Table 3 summarizes representative in vitro, animal, and human studies included in this review on the biological effects of honey phenolic-derived microbial metabolites, including study models, interventions, and key outcomes.

## 7. Conclusions and Future Prospects

### 7.1. Core Conclusions

The systematic integration of the available evidence yields three principal inferences, which must be interpreted with due caution given the nature of the current evidence base.

First, the health benefits of honey phenolic compounds are not primarily attributable to the intact parent molecules, but are largely mediated by, and strongly associated with, the microbial metabolites generated through gut microbiota metabolism. Although the plasma concentrations of native phenolic precursors are extremely low (in the nanomolar range), their microbiota-derived small-molecule metabolites can reach biologically relevant levels both locally in the intestinal lumen and systemically. This observation supports a working model in which microbial metabolites serve as the primary effectors, rather than the original phenolic glycosides or aglycones.

Second, the intestinal microbiota transforms honey phenolic compounds through a sequential, multistep biotransformation pathway—namely deglycosylation, C-ring cleavage, and reduction/secondary modification—which collectively enhances the absorbability and bioactivity of the resulting metabolites. However, it should be noted that the precise flux and regulatory nodes of this pathway in the human intestine remain largely inferred from ex vivo and animal models, and await direct in vivo validation.

Third, the generated microbial metabolites may exert pleiotropic regulatory effects on host metabolism, neuroinflammation, immune homeostasis, and energy balance, potentially involving the gut–liver, gut–brain, gut–immune, and gut–fat axes. Nevertheless, the causal relationships and physiological relevance of these multi-target effects in humans remain speculative, as most evidence derives from mechanistic models rather than from human interventional studies.

Crucially, a fundamental limitation of the current evidence base must be explicitly acknowledged: the vast majority of findings summarized in this review are derived from in vitro fermentation models, animal studies, or ex vivo human fecal cultures. While these models provide mechanistic insights and are indispensable for hypothesis generation, they cannot fully replicate the complex physiological environment of the human intestine, including dynamic flow, mucus layer, immune interactions, and inter-individual microbial variability. Randomized controlled trials in healthy populations and patient groups are extremely limited, making it difficult to support causal inference and dose recommendations.

### 7.2. Key Issues to Be Addressed

The explicit acknowledgment of the current evidence limitations naturally leads to the identification of several interconnected knowledge gaps that must be resolved to advance the field.

(1) Insufficient human evidence and limited causal inference. As highlighted in Section 7.1, the most fundamental barrier to translational application is the severe shortage of high-quality human intervention studies. The overwhelming majority of data available to date derive from in vitro fermentation systems, animal models, or ex vivo fecal cultures. These approaches, while valuable for mechanistic exploration, cannot account for the full complexity of the human intestinal ecosystem, including dynamic fluid flow, mucus layer integrity, immune–microbial crosstalk, and the substantial inter-individual variability in microbial composition and metabolic capacity. Consequently, well-designed randomized controlled trials in both healthy volunteers and relevant patient populations are urgently needed to establish causal relationships between honey phenolic metabolism and clinical outcomes, and to derive evidence-based dosing regimens.
(2) Multi-component synergy and network complexity. Honey is not a simple phenolic extract but a chemically intricate matrix containing hundreds of constituents—including diverse phenolic glycosides, oligosaccharides, enzymes, and unique specialized metabolites such as methylglyoxal (in Manuka honey) or defensin-1. How these components collectively influence the gut microbial metabolism of phenolic compounds, and whether they act synergistically, additively, or antagonistically, remains poorly understood. Deciphering this multidimensional interaction network is a prerequisite for understanding the integrated health effects of honey beyond its isolated phenolic fractions.
(3) Lack of standardized metabolic signatures. A major obstacle to cross-study comparison and clinical translation is the pronounced heterogeneity in experimental designs, including variations in honey types, dosages, intervention durations, and analytical platforms. Currently, there is no universally accepted panel of characteristic microbial metabolites—a “metabolic signature”—that can serve as a standardized quality marker for metabolite-based honey products or as a benchmark for evaluating their prebiotic efficacy. The development of such signatures is critical to enable reproducible research, facilitate meta-analyses, and support regulatory standardization.
(4) Unpredictable inter-individual variability. Substantial differences exist among individuals in the metabolic profiles and health outcomes following honey consumption, yet the ability to predict who will derive benefit and who will remain non-responsive is currently lacking. This heterogeneity arises from multiple interacting determinants, including gut enterotypes, baseline β-glucosidase gene copy numbers within the microbiome, host genetic polymorphisms, and dietary background. Translating these complex interactions into a practical, clinically applicable prediction model remains an unmet challenge.

Taken together, these gaps are not mutually exclusive but are deeply intertwined, collectively impeding the transition from observational and mechanistic findings to actionable nutritional interventions.

### 7.3. Future Research Directions

Addressing the above-mentioned challenges will require a coordinated, multidisciplinary effort that spans fundamental microbiology, analytical chemistry, clinical nutrition, and bioinformatics. The following four strategic directions are proposed.

(1) Well-designed human intervention trials. The highest priority is to conduct rigorous randomized controlled trials in well-characterized human populations, using standardized honey preparations and clearly defined phenolic metabolite profiles as both compliance and effect markers. Future trials should move beyond measuring plasma parent compounds as primary endpoints, and instead adopt fecal and circulating metabolite spectra as the main outcome measures, assessed via targeted or untargeted metabolomics. These metabolic readouts should be correlated with functional physiological endpoints—such as insulin sensitivity, cognitive performance, colitis activity indices, or inflammatory biomarkers—to directly test the causal chain from honey intake → microbial metabolism → metabolite production → host response.
(2) Systems-level deciphering of the honey–microbiome interactome. Advanced multi-omics approaches—integrating metagenomics, metatranscriptomics, metabolomics, and culturomics—should be employed to unravel the synergistic networks among honey components and the gut microbial ecosystem. In particular, co-culture systems using defined bacterial consortia, combined with stable isotope tracing, can help distinguish which specific components drive metabolic flux through the phenolic transformation pathway. This systems-level understanding is essential for rationally designing honey-based synbiotic combinations.
(3) Establishment of standardized metabolite-based quality markers. A collaborative effort is needed to define a consensus panel of characteristic microbial metabolites derived from honey phenolic fermentation, which could serve as a robust and reproducible quality indicator. This signature should be validated across different honey varieties, laboratories, and populations. Once established, it could function as a benchmark for product standardization, facilitate regulatory approval, and enable head-to-head comparisons across future clinical studies.
(4) Development of precision nutrition algorithms. To address inter-individual variability, future research should integrate host factors (e.g., gut enterotype, baseline enzymatic activity, genetic background, age, and dietary habits) into predictive machine-learning models that stratify individuals into “responder” versus “non-responder” categories. Such algorithms would enable personalized honey recommendations, optimizing both the choice of honey type and the effective dosage for each individual. This vision will require large-scale cohort studies with longitudinal sampling and robust bioinformatics pipelines.

Ultimately, concerted efforts in these directions will not only strengthen the mechanistic and clinical evidence base but also pave the way for the transformation of honey from a traditional food into a precisely targeted dietary intervention for metabolic, inflammatory, and cognitive health.

## 8. Literature Review Methodology

This review adopts a narrative approach to systematically summarize the intestinal metabolic patterns of honey phenolic compounds and their prebiotic effects. Literature searches were conducted in Web of Science, PubMed, and Scopus databases, covering publications from 1996 to 2026. The search terms included combinations of “honey,” “phenolic compounds,” “polyphenols,” “flavonoids,” “gut microbiota,” “intestinal metabolism,” “bioavailability,” “prebiotic,” “fermentation,” “hepatoprotection,” and “neuroprotection.” Inclusion criteria focused on peer-reviewed original research and reviews addressing the metabolism, bioavailability, or biological activities of honey phenolics mediated by gut microbiota, and did not require that journals be indexed in the Science Citation Index Expanded. Exclusion criteria were non-English articles, conference abstracts, and studies unrelated to gut microbial metabolism. Literature screening and critical appraisal were independently performed by two authors, with disagreements resolved through discussion and consensus.

## Figures and Tables

**Table 1:** Comprehensive comparison of phenolic composition, structural–metabolic determinants, and metabolite profiles of honeys from different floral origins.

Honey Source	Characteristic Phenolics and Main Components	Marker Compounds/Features	Key Gut Microbial Substrate/Pathway	Key Structural Mechanisms Determining Metabolite Generation	Major Microbial Metabolites and Microbiota Shifts	References
Buckwheat honey	Phenolic acids: fumaric acid, p-hydroxybenzoic acid, p-coumaric acid, trans-2-hydroxycinnamic acid; Flavonoids: rutin, quercetin (major), catechin; Oligosaccharides: kestose, isomaltose, panose	4-Hydroxybenzaldehyde, 2-hydroxypyridine, hydroxyquinoline-3-carbonitrile	Rutin-3-O-glucoside/β-glucosidase-mediated deglycosylation → C-ring cleavage by *Eubacterium ramulus*	C2 = C3 bond triggers *E. ramulus* cleavage; 3-O-glucoside is quickly deglycosylated by β-glucosidase; retained B-ring ortho-dihydroxyls then produce 3,4-DHPAA.	3,4-DHPAA (12.3 ± 1.8 µM), 3,4-DHPPA, butyrate; ↑ *Bifidobacterium*, ↑ *Lactobacillus*, ↑ *E. ramulus;* ↓ opportunistic pathogens	[10,40,47,49,60]
Citrus honey	Flavanones: hesperidin, hesperetin, naringenin; Phenolic acids: caffeic acid, p-coumaric acid, p-hydroxybenzoic acid	Hesperetin (reliable chemical marker for citrus honey)	Hesperidin-7-O-glucoside/β-glucosidase → saturated C-ring reduction/cleavage by *Flavonifractor plautii*	Saturated C-ring (no C2 = C3) is not used by E. ramulus but is cleaved reductively by F. plautii; the retained C6-C3 skeleton then gives 3-phenylpropionic acid derivatives.	3-Phenylpropionic acid derivatives (8.7 ± 0.9 µM), acetate, propionate, butyrate, valerate; ↑ Bacteroidetes phylum; ↓ Firmicutes phylum, ↓ *Turicibacter* genus	[61]
Manuka honey	Phenolic acids: syringic acid, 4-methoxyphenyllactic acid, 4-hydroxybenzoic acid, benzoic acid, 2-methoxybenzoic acid; Others: methylglyoxal (MGO), kojic acid, dehydrovomifoliol, leptosin	Group 1: 4-hydroxybenzoic acid, benzoic acid, dehydrovomifoliol; Group 2: kojic acid, 2-methoxybenzoic acid; Group 3: syringic acid, 4-methoxyphenyllactic acid	Free phenolic acids (non-glycosidic)/side-chain β-oxidation (no C-ring cleavage required)	No C-ring or glycosylation bypasses the two rate-limiting steps (deglycosylation and C-ring cleavage); free acids directly undergo β-oxidation to methylated small phenolic acids, giving the shortest route and simplest profile	Methyl syringate, dihydrocaffeic acid (4.2 ± 0.6 µM), short-chain fatty acids; modulates neutrophil release profile (MPO release reduced by ~70% at 1300 µM)	[62,63]
Heather honey (Erica spp.)	Phenolic acids: chlorogenic acid, p-coumaric acid; Flavonoids: kaempferol, myricetin derivatives; Volatiles: hotrienol (up to 69%), phenylacetaldehyde, cis-linalool oxide	Hotrienol (clear marker for all heather honeys, unaffected by species)	Arbutin, ellagitannins/β-glucosidase → hydrolysis of glycosidic bonds → aromatic ring reduction	Arbutin deglycosylates to hydroquinone directly (no C-ring cleavage). Ellagitannins are esterase-hydrolyzed, then spontaneously open and decarboxylate to urolithin A/B. The generation of these metabolites depends on esterase rather than glycosidase activity.	Hydroquinone, urolithin A (6.5 ± 1.1 µM), propionate, butyrate; ↑ *Lactobacillus*, ↑ *Bifidobacterium*; stimulates probiotic metabolism	[60,64]
Acacia honey	Flavonoids: pinobanksin (highest content), chrysin, galangin, quercetin, hesperetin; Phenolic acids: p-hydroxybenzoic acid	Quercetin, p-syringaldehyde (marker compounds for acacia honey)	Pinobanksin-7-O-glucoside/β-glucosidase → limited C-ring cleavage (low affinity for *Flavonifractor* substrates)	7-O-Glucoside has weaker β-glucosidase affinity than the 3-O-type, slowing deglycosylation and aglycone supply; pinobanksin, though saturated in the C-ring, lacks B-ring catechol, further reducing cleavage enzyme recognition; thus, overall flux is very low.	Phenylpropionic acid derivatives (limited yield, estimated 2–4 µM); ↑ *Bifidobacterium* (moderate); insufficient evidence for deep ring cleavage	[34,65,66]
Strawberry tree honey (*Arbutus unedo*)	Homogentisic acid (HGA), ellagic acid, gallic acid, kaempferol, arbutin; (±)-2-cis,4-trans-abscisic acid (c,t-ABA), (±)-2-trans,4-trans-abscisic acid (t,t-ABA), unedone	Homogentisic acid (most reliable marker), ABA and the new compound unedone	Ellagitannins, gallotannins, arbutin/gut microbial esterase and β-glucosidase → lactone ring opening	Ellagitannins, esterase-hydrolyzed, rearrange/decarboxylate to urolithins (no C-ring cleavage). Arbutin (like heather honey) releases hydroquinone. The two substrates give a profile of both urolithins and hydroquinone.	Urolithin A/B, p-hydroxybenzoic acid, catechol, hydroquinone (urolithins: 6.5 ± 1.1 µM); ↑ *Lactobacillus*, ↑ *Bifidobacterium* (strong stimulation in vitro)	[64,67]

↑ indicates increased abundance/growth promotion, and ↓ indicates decreased abundance/growth inhibition.

**Table 2:** Phenolic compounds, gut microbiota metabolism, and health effects of honeys from different floral sources.

Honey Source	Characteristic Phenolic Compounds	Major Gut Microbiota Metabolites	Key Metabolic Genera/Gut Microbiota Changes	Potential Health Effects	Reference
Citrus honey	Hesperetin, hesperidin, chlorogenic acid, p-hydroxybenzoic acid, caffeic acid	Deglycosylation releases hesperetin aglycone, generates small-molecule phenolic acids; increases acetate, propionate, butyrate, valerate	Increases *Bacteroidetes* phylum abundance, suppresses *Firmicutes* phylum, *Campylobacterota* and *Turicibacter* genus	Reduces AST and ALT levels in mice with alcoholic liver disease, alleviates hepatocyte edema and histopathological liver injury	[61]
Buckwheat honey	Quercetin, 4-hydroxybenzoic acid, p-coumaric acid; naturally co-existing oligosaccharides (kestose, isomaltose, panose, etc.)	Quercetin degradation to 3,4-DHPAA by *E. ramulus*; butyrate and other SCFA from oligosaccharide fermentation	*Eubacterium ramulus*, *Bifidobacterium* spp., *Lactobacillus* spp. *Bacteroides* spp.	Oligosaccharides and phenolic substrates constitute a coordinated metabolic system, enhancing phenolic metabolic capacity of gut microbiota; selectively promotes beneficial bacteria such as *bifidobacteria*, inhibits intestinal pathogens	[10,40,47,49,60]
Honeydew honey	Total phenolics 806.10 mg GAE/kg, total flavonoids 146.27 mg QE/kg	Deglycosylation of phenolics releases aglycones; oligosaccharides and phenolics synergistically promote SCFA (acetate, propionate, butyrate) production	*Bifidobacterium* spp., *lactic acid bacteria* (LAB); microencapsulated honeydew honey resists gastric acid and digestive enzymes for targeted colonic delivery	Promotes probiotic proliferation and delays decline phase; microencapsulation enhances adhesion and colonization potential of probiotics in the gastrointestinal tract	[68]
Strawberry tree honey (*Arbutus unedo* L.)	Homogentisic acid (HGA), ellagic acid, gallic acid, kaempferol, arbutin	Human gut microbiota ferment ellagitannins, gallotannins, flavan-3-ols and anthocyanins to produce small-molecule phenolic acids such as p-hydroxybenzoic acid and catechol	Human gut microbiota (strains to be identified); stimulates the growth of probiotic strains such as *Lactobacillus* and *Bifidobacterium*	Extremely high phenolic content confers strong antioxidant activity; in vitro evidence of positive stimulation on probiotic metabolism; antimicrobial, anti-cholesterol lipid peroxidation activity	[64]

**Table 3:** Summary of in vitro, animal, and clinical evidence for honey phenolic microbial metabolites.

Study Type	Model/Sample	Intervention/Metabolite	Key Quantitative Findings	Ref.
In vitro	SHIME^®^ dynamic colon model	Honey oligosaccharides + phenolic mixture	Butyrate by 7 mmol/L in ascending colon; Acetate/Propionate/Butyrate by ~4 mmol/L in transverse/descending colon; *Lactobacillus* & *Bifidobacterium* by ~2.5 Log/mL.	[27]
In vitro	Human PBMCs (LPS-stimulated)	Microbial metabolites (3,4-DHPAA/3,4-DHPPA)	TNF-α secretion inhibited by 84.9%/86.4%; IL-6 reduced by 88.8%/92.3%.	[7]
In vitro	Human neutrophils (PMA-stimulated human neutrophils)	Methyl syringate (Manuka honey metabolite)	Dose-dependent (600–1300 µM) inhibition; MPO release reduced by ~70% at 1300 µM; IL-8 suppression peaked at 6 h (returned to baseline).	[62]
In vitro	Co-culture (*E. ramulus* + *B. thetaiotaomicron*)	Starch + Quercetin	Starch hydrolysis by *B. thetaiotaomicron* significantly enhanced quercetin degradation by *E. ramulus* (qualitative enhancement).	[47]
Animal	Rat NASH model (High-cholesterol diet)	Tualang honey (1.2 g/kg/day, oral)	Serum ALT: 77.25 → 50.25 U/L (*p* = 0.025); AST: 193.25 → 106.75 U/L (*p* < 0.01); Histological NAS significantly improved.	[84]
Animal	Mouse DSS-induced colitis	Protocatechuic acid (5 or 25 mg/kg, 15 days)	Dose-dependently restored ZO-1/claudin-2/4 distribution; 25 mg/kg superior in reducing colonic IL-1β & TNF-α mRNA.	[94]
Animal	Rat (Cecal microbiota)	Fructo-oligosaccharides (FOS) + Polyphenol concentrate (PC)	Enhanced the adaptive response of the cecal microbial enzymatic system	[28]
Animal	Antibiotic-treated mice	Tea polyphenols (EGCG, etc.)	Significant accumulation of intact EGCG in serum/liver; intact catechins detected in urine.	[77]
Human (ex vivo)	Fecal fermentation (Different age groups)	Ferulic acid	Degradation efficiency and conversion rates significantly reduced in the elderly population (qualitative).	[76]
Human (Cohort)	Crohn’s disease patients vs. Healthy controls	Dietary polyphenols/Fecal microbiome	Patients showed significantly impaired flavonoid-degrading capacity; Serum hippuric acid (metabolite marker) markedly decreased.	[78]

## Data Availability

No new data were created or analyzed in this study. Data sharing is not applicable to this article.
